# Determining paracrystallinity in mixed-tacticity polyhydroxybutyrates

**DOI:** 10.1107/S1600576720015794

**Published:** 2021-02-01

**Authors:** Daniel Van Opdenbosch, Maria Haslböck, Cordt Zollfrank

**Affiliations:** a Biogenic Polymers, Technical University of Munich, Campus Straubing for Biotechnology and Sustainability, Schulgasse 16, D-94315 Straubing, Germany

**Keywords:** polyhydroxybutyrates, mixed tacticity, paracrystallinity, Rietveld refinement, thermal factors

## Abstract

Recently, the authors reported on the development of crystallinity in mixed-tacticity polyhydroxybutyrates. Comparable values reported in the literature vary depending on the manner of determination, the discrepancies being partially attributable to scattering from paracrystalline portions of the material. These portions can be qualified by peak profile fitting or quantified by allocation of scattered X-ray intensities. However, the latter requires a good quality of the former, which in turn must additionally account for peak broadening inherent in the measurement setup, and due to limited crystallite sizes and the possible presence of microstrain. Since broadening due to microstrain and paracrystalline order both scale with scattering vector, they are easily confounded. In this work, a method to directionally discern these two influences on the peak shape in a Rietveld refinement is presented. Allocating intensities to amorphous, bulk and paracrystalline portions with changing tactic disturbance provided internal validations of the obtained directional numbers. In addition, the correlation between obtained thermal factors and Young’s moduli, determined in earlier work, is discussed.

## Abstract 2

A method to robustly determine paracrystalline contents from Rietveld-refined powder X-ray data is presented and discussed for the example of mixed-tacticity polyhydroxybutyrates.

## 1. Introduction   

### 1.1. An open question with regard to mixed-tacticity polyhydroxybutyrates   

Polyhydroxybutyrates (PHBs) are thermoplastic polymers that are synthesized by various microorganisms for energy storage (Lundgren *et al.*, 1965 ▸). They are renewable by biosynthesis, biodegradable and thermally processable, a combination that makes them of technological interest. In nature, exclusively isotactic PHB is produced from *R*-3-hydroxybutanoic acid and crystallizes in the α-PHB structure (Okamura & Marchessault, 1967 ▸; Barham *et al.*, 1984 ▸; Wang & Tashiro, 2016 ▸). On the basis of earlier work (Bloembergen *et al.*, 1989 ▸; Abe *et al.*, 1994 ▸; Kemnitzer *et al.*, 1995 ▸), we recently prepared synthetic variants in which defined amounts of *S* monomers were included randomly into the polymer chains (Haslböck *et al.*, 2018 ▸). By nuclear magnetic resonance spectroscopy, we determined the respective compositions’ fractions of meso diads *f*_meso_ to be quadratically correlated to the fractions of *R* monomers *f*_R_. Since the *f*_meso_ values can be accurately determined in the final material, we used them for quality control and as the characterizing numbers for the different compositions.

An application-oriented aim of the overall venture was to determine the progressions of structural, mechanical and thermal characteristics with *f*_meso_. Hence, in two prior studies, we determined the progression of the fraction of crystalline phase and the accompanying crystallite sizes with *f*_meso_ (Haslböck *et al.*, 2018 ▸) and some mechanical and thermal properties of the compositions (Haslböck *et al.*, 2019 ▸). The latter included glass transition temperatures, which remained constant at 278 K, and melting points, which decreased linearly from 440 K at *f*_meso_ = 1.00 to 330 K at *f*_meso_ = 0.59, below which no melting signals were detected.

Furthermore, we attempted to find a characteristic that would account for observed anomalies in the progressions of various properties, at least qualitatively (Haslböck *et al.*, 2019 ▸). We used a random-number generator to simulate *R* and *S* monomer chain makeups and then fitted the iso- and atactic chain segment lengths with exponential decay functions. From the decay coefficients λ for the iso- and atactic components, we calculated the number 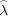. This is a qualitative indicator of chain disturbance due to mismatching tacticities and peaks at the intersect of the two λs, which is at *f*_meso_ = 0.7 for randomly added monomers. It is there that instances of unexpected mechanical and thermal behaviour were detected (Haslböck *et al.*, 2019 ▸).

But there was an issue to be resolved: We reported on fractions of crystalline phase *f*_c_ that decreased with decreasing *f*_meso_, but remained above 0.6 down to *f*_meso_ = 0.64. Then, we scaled a diffractogram recorded from a rapidly regenerated thin sheet of PHB to obtain the amorphous phase intensities [Fig. 1 of Haslböck *et al.* (2018 ▸)]. The same approach, using ice-water-quenched PHB to record an amorphous phase diffractogram, was used by Bergmann & Owen (2003 ▸). Comparing the obtained values with an infrared spectroscopy-based index *I*_c_ yielded a reasonably linear correlation [Fig. 10 of Haslböck *et al.* (2018 ▸)]. We were further able to trace the melting enthalpies Δ*H*_m_ of our materials by the Gibbs–Thompson relation, suggesting the correctness of the *f*_c_ values [Fig. 11 of Haslböck *et al.* (2019 ▸)]. However, the development of *f*_c_ to lower *f*_meso_ remained unclear, in particular to *f*_meso_ = 0.54, of which we had obtained an X-ray diffractogram [Fig. 6 of Haslböck *et al.* (2018 ▸)]. Extrapolating the fitted curve of *f*_c_ (*f*_meso_) to *f*_meso_ = 0.54 would give a value of *f*_c_ = 0.57 ± 0.16, whereas the diffractogram did not show any appreciable Bragg reflections. Similarly, by extrapolating the progression of Δ*H*_m_, one would expect a value of ∼10 J g^−1^ at *f*_meso_ = 0.54, whereas no melting signal was observed. We felt that this issue of ‘abruptly missing crystalline phase’ might be resolved by taking into account the material fraction exhibiting paracrystalline order.

### 1.2. Paracrystallinity in polymers   

Materials whose X-ray diffractograms can be accounted for as originating from structures exhibiting disturbed crystalline and fluid statistical order, or, conversely, disorder of the second kind (the first kind being thermal disorder) are termed paracrystalline, a concept coined by Rinne (1931 ▸, 1932 ▸, 1933 ▸) and expanded by Hosemann (Hosemann, 1950 ▸; Hosemann & Wilke, 1968 ▸). Rinne pointed out the origin of the term (Lehmann, 1918 ▸) and sharpened the nomenclature, distinguishing paracrystals from liquid crystals (the former being solids), and other proposed names such as mesomorphous or pseudocrystals (Rinne, 1933 ▸). However, terms such as ‘mesomorphous smectic’ or ‘with smectic character’ remained in use, alluding to structuring also found in liquid crystals (Natta & Corradini, 1960 ▸). To not count as amorphous, paracrystals must possess lattice characteristics, such as a fixed number of structural neighbours. However, in contrast to undisturbed crystals (to which we apply the adjective ‘bulk’, for better differentiation and to stress their long-range order in all directions), paracrystals may incorporate structural disturbances that are not systematically transferred to their neighbours, resulting in the loss of long-range order (De Rosa & Auriemma, 2013 ▸).

Early on, it was recognized that polymers are likely to exhibit paracrystallinity (Hosemann, 1950 ▸). Along their chains, atomic positions are defined by the repeating unit periodicity, while perpendicularly there may be freedom of movement (Natta & Corradini, 1960 ▸). Combined with the diverse possibilities of intra- and intermolecular interactions, paracrystallinity may present itself in a large variety of structural arrangements (Hosemann, 1963 ▸, 1970 ▸). Miller described this structural organization in polymers, which he termed ‘non-crystalline’, as ‘neither amorphous nor crystalline and […] very stable, for only slight changes in the X-ray scattering curve were detected even after the sample had been stored at room temperature for one and a half years’ (Miller, 1960 ▸).

A prominent case of paracrystallinity can be found in polymers that exhibit folding, such as polyethylene and PHB (Hosemann, 1963 ▸, 1970 ▸; Hosemann & Wilke, 1968 ▸). Here, ‘[t]he paracrystalline concept takes into account that each ‘crystalline’ lamella consists of a series of microparacrystallites with grain boundaries in between them’ (Hosemann, 1975 ▸). Hosemann also included molecular aggregate superstructures (Hosemann, 1950 ▸) into the definition (sand patterns on beaches, as well; Hosemann, 1963 ▸), although here, the length scale of the paracrystalline order exceeds that accessible to X-ray diffraction.

Paracrystalline regions may be considered structural features – defects – of crystalline arrangements. However, as also outlined above, regions that exhibit paracrystalline order are interdispersed with undisturbed crystalline regions, as are regions showing amorphous order. Furthermore, they exhibit distinct properties. To trace these, they are sensibly treated as separate, intermediate phases (Wada *et al.*, 1967 ▸; De Rosa & Auriemma, 2013 ▸). There are two general methods to investigate paracrystalline order: directionally and qualitatively by resolving Bragg peak broadening contributions, and quantitatively by allocating intensities of diffractograms.

### 1.3. Qualification of paracrystalline order from Bragg peak broadening   

A directional assessment of paracrystalline order considers Bragg peak broadening. The contributions to the directional integral peak width β due to average crystallite size 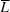, microstrain-derived lattice parameter distribution ϕ^−1^(ε) and a measure of paracrystalline order *g* can be distinguished by their progressions with scattering vector *s* = *n*/*d* for the lattice plane distances *d* and the reflection scattering orders *n* (Hosemann & Wilke, 1968 ▸):
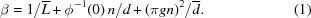
Prior to the widespread availability of the Rietveld refinement method (Rietveld, 1969 ▸), Williamson–Hall plots (Williamson & Hall, 1953 ▸) were used to separate the broadening contributions due to crystallite size and strain. Similarly, linear fits to directional plots of peak width over squared scattering order allowed one to calculate *g* by equation (2):
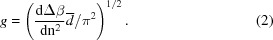
Here, ϕ^−1^(0) is the breadth of the microstrain distribution function, equal to half the apparent strain ε = Δ*d*/*d* (Stokes & Wilson, 1944 ▸). 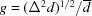 is a measure of the amount of lattice distortion relative to the distortion-averaged lattice plane distances 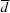, ranging from 0 for perfect crystals to 1 for ideal gases (Hosemann & Hindeleh, 1995 ▸). If expressed directionally in terms of the Miller indices {*hkl*}, *g* provides a measure of paracrystalline order between lattice planes for a given direction.

### 1.4. Quantification of phase contents by allocating X-ray diffractometry intensities   

Since the aforementioned broadening is accompanied by a loss of Bragg peak intensity (Hosemann, 1950 ▸; Ruland, 1961 ▸) one can designate a portion of the scattered, but not Bragg-diffracted, intensity as stemming from paracrystalline domains. The remainder of the coherent background is due to the amorphous phase and thermal diffusive scattering. Depending on the measurement setup, incoherent scattering may also be detected, requiring intensity corrections (Ruland, 1961 ▸). In agreement, Mu (1998 ▸) demonstrated that scattering from paracrystals attenuates quickly into a continuous background, the intensity of which progresses by a function similar to that for disorder of the second kind cited by Vonk (1973 ▸).

An example with direct relevance for the present work was given by Hosemann, separating the previously assigned (Hermans & Weidinger, 1948 ▸) amorphous portion of scattering from cellulose materials into amorphous and paracrystalline fractions [Fig. 4 of Hosemann (1950 ▸)]. In individual polymer materials, the fraction exhibiting paracrystalline order can be significant; for example, quantification of polytetrafluoroethylene via dilatometry yielded a value of the paracrystalline phase content *X*_p_ = 0.48, exceeding that of the bulk crystalline phase, *X*_c_ = 0.45 (Ohzawa & Wada, 1964 ▸).

In Ruland’s (1961 ▸) words, the intensities of the ‘crystalline peaks’ and the ‘amorphous background’ cannot be unambiguously correlated with the weight fractions of crystalline and amorphous material, since ‘even an entirely crystalline substance shows diffuse coherent scattering and a loss in intensity of the diffraction peaks due to thermal vibrations of the atoms as well as to lattice imperfections, effects which have been emphasized in a recent paper by Hosemann *et al.* (1960) (sic)’ [referencing Bonart *et al.* (1960 ▸)]. Therefore, ‘some structural details are needed to evaluate the crystallinity, or more correctly, the amount of material which shows two- or one-dimensional order […]’ (Ruland, 1961 ▸). On this basis, and in homopolymers, the determination of bulk crystalline phase fractions by X-ray diffractogram integration over the volume of reciprocal space is performed by equation (3) (Ruland, 1961 ▸):
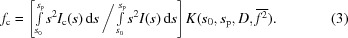
Here, 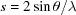 is the scattering vector as a function of the scattering angle θ and the wavelength λ, and *I* and *I*_c_ are the total scattered intensity and the portion thereof within the diffraction peaks, respectively. *K* is a correction function, wrapping the disorder function *D*. *s*_0_ and *s*_p_ are the integration boundaries and 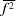 is the squared number-weighted sum scattering factor of all atoms within the unit cell. *D* can be first-approximated by the thermal Debye–Waller factor (Ruland, 1961 ▸; Vonk, 1973 ▸).

In the original method, as expanded by Vonk (1973 ▸), the intensities within the Bragg peaks, separated by a simulated continuous background, were considered. Then, for materials containing large degrees of structural disorder, the cumulative integral fractions as functions of *s*_p_ follow curves, rather than straight lines, when applying the Debye–Waller factor. In this case, equation (3) can (is intended to) account for paracrystallinity, by modifying *D* (Ruland, 1964 ▸). However, this leads to the follow-up issue of selecting suitable disorder functions, accounting for thermal motions, microstrain and paracrystallinity, not only for specific crystal structures (Kavesh & Schultz, 1969 ▸) but also for individual crystallographic directions (Ruland, 1961 ▸). Worded one way or another, researchers have found the separation of contributions to be the major obstacle in applying equation (3) (Kavesh & Schultz, 1969 ▸; Mo & Zhang, 1995 ▸; Vonk, 1973 ▸).

Subsequent to Ruland’s formulation, Rietveld developed his full-profile refinement method (Rietveld, 1969 ▸). Given the right structural model and suite of parameters, including the crystallite sizes and thermal and/or structural disorder, Rietveld refinement will result in values of the scattered intensities within the Bragg peaks, which thitherto were determined graphically (Kavesh & Schultz, 1969 ▸; Mo & Zhang, 1995 ▸; Vonk, 1973 ▸). Then, considering the intensities transferred to the continuous background by disorder allows for a separation from the remaining background (Riello, 2004 ▸, Section 7.3 in the cited book chapter). Using refined values of *I*_c_ and adding the portion of the background due to the accompanying disorder removes the necessity to account for disorder separately via *K*. Then, equation (3) converges on the value of *f*_c_ with increasing *s*_p_ – in principle.

In practice, however, Rietveld refinement requires good models for all phases in order to provide robust results, including the background function: ‘If the polynomial happens to describe the background well, then, as might be expected, [refining the background] works well […]’ (McCusker *et al.*, 1999 ▸), noting the caveat ‘happens to’. Riello concluded the same, namely that one should apply a diffractogram from a pure amorphous material, multiplied by a scale factor (Riello, 2004 ▸). Alternatively, if the smooth background belonging to the crystalline phases is properly taken into account, ‘the amorphous contribution can be fairly well described by using a polynomial or other more suited functions’ (Riello, 2004 ▸), noting the qualifier ‘fairly’.

So, conceptually, Rietveld refinement works ‘downwards’ from the crystalline structure and the Bragg peaks, whereas separation via Ruland and Vonk’s method works ‘upwards’, from the continuous background. These two approaches were distinguished by Riello (2004 ▸) in a review of methods. He further pointed out the method of calculating calibration lines from reference samples with known amounts of amorphous phase, as well as our earlier (Haslböck *et al.*, 2018 ▸) approach (‘limiting the analysis to the 2θ range which corresponds to the first halo of the amorphous pattern’) (Riello, 2004 ▸).

### 1.5. Simultaneous considerations   

It was the main aim of this work to determine and verify the lattice orientations and amounts of paracrystalline order in mixed-tacticity polyhydroxybutyrates with changing *f*_meso_, by applying the aforementioned qualitative and quantitative approaches simultaneously. This required a Rietveld routine able to identify paracrystalline order among other contributions to peak broadening, as well as knowledge of the scattering profiles from the amorphous and paracrystalline fractions.

We determined a method for distinguishing microstrain and paracrystalline order in a Rietveld refinement. To refined patterns, we applied equation (3), using the convergences of cumulative integrals with increasing *s*_p_ on their final values as a means to attribute attenuated Bragg peak intensities. In this equation, we substituted the index *f*_c_ for *f*_*i*_, for any *i* of the amorphous (am), bulk (bc) and paracrystalline (pc) phases.

The secondary aim was to correlate obtained structural information to previously determined structural (Haslböck *et al.*, 2018 ▸), mechanical and thermal (Haslböck *et al.*, 2019 ▸) properties.

## 2. Experimental   

### 2.1. Polymer synthesis   

Details of the polymer synthesis were reported earlier (Haslböck *et al.*, 2018 ▸). In brief, *R*- and *S*-β-butyrolactone monomers were polymerized with defined fractions of *R*, *f*_R_ > 0.5. We utilized ethylzinc β-diketiminate as catalyst and 4-methoxybenzyl alcohol as co-catalyst in dry toluene under stirring for 24 h at 353 K, using standard Schlenk techniques. The reactions were quenched with small amounts of methanol, ground with a mortar and washed with methanol. All materials were used as synthesized and after ageing for at least seven days.

From nuclear magnetic resonance spectroscopy, we determined the fraction of *meso* diads *f*_meso_ for each prepared material. Since they are fixed properties of the PHB chains themselves, they were subsequently used as their descriptor. Owing to the spacing between the assessed *f*_meso_, characteristic values or ranges are naturally approximates with an uncertainty given by the distance between adjacent data points. Hence, any statement ‘*f*_meso_ (<, =, >) *x*’ means ‘
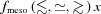
’, with the aforementioned uncertainty.

### 2.2. X-ray diffractometry   

The data used in this work were obtained from a powder diffractometer (Miniflex, Rigaku, Tokyo, Japan) with a copper anode and a silicon strip detector (D/teX Ultra, Rigaku) and have been reported on elsewhere (Haslböck *et al.*, 2018 ▸). The setup in detail: goniometer radius 150 mm; both Soller slits 2.5°; divergence slit fixed at 0.625°, but closing variably below 10° 2θ; anti-scatter slit 8 mm; no monochromator; *K*β filter 0.06 mm nickel foil; effective receiving slit of the multiline detector 0.1 mm. Two additional measurements were made from samples with *f*_meso_ = 0.54 and *f*_meso_ = 0.6. In total, 86 measurements were performed, spread over the range of *f*_meso_ in groups of two to ten samples per reported data point. The data were evaluated by Rietveld refinement (*BGMN*, using the *Profex* interface) (Bergmann *et al.*, 1998 ▸; Bergmann & Taut, 2005 ▸; Doebelin & Kleeberg, 2015 ▸) considering the machine line function, as verified by refining NIST standards 640e and 660c (silicon and lanthanum hexaboride for peak shapes and positions; machine parameter file and refinement results available from the authors). The scattering angle range 5 < 2θ < 75° was considered and the sample offset from the goniometer axis refined. Reflections from the holders, occurring in the ranges 36.2 < 2θ < 37.2°, 42.4 < 2θ < 43.4° and 62.9 < 2θ < 63.9°, were excluded.

We based the bulk and paracrystalline phases on the α-PHB structure with orthorhombic space group 19 and lattice parameters *a* = 0.573 nm, *b* = 1.315 nm and *c* = 0.593 nm (Wang & Tashiro, 2016 ▸). Being in the orthorhombic crystal system, and only considering directions normal to the unit-cell sides, we freely equate the normals of the lattice plane families {100}, {010} and {001} with the directions of the lattice parameters *a*, *b* and *c*, for simplicity. Since we are considering statistical averages, the actual lattice parameter dimensions are denoted 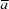, 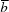 and 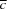.

**Figure 1:**
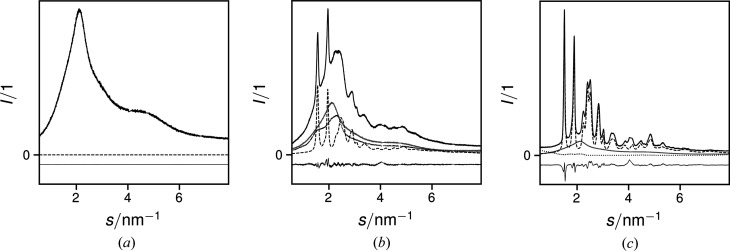
Recorded diffractograms (black solid curves), as deconvolved into amorphous background (grey solid curves), scattering from bulk crystals (dashed curves) and paracrystalline scattering (dotted curves) of samples with (*a*) *f*_meso_ = 0.54, (*b*) *f*_meso_ = 0.64 and (*c*) *f*_meso_ = 1.00, together with the refinement residuals (thin curves at negative values). In (*a*), scattering from non-amorphous phases is 0.

The diffractogram from a sample with the lowest *f*_meso_, presented in Fig. 1(*a*), was taken as the scattering profile of the amorphous fraction, noting its agreement with one measured by Bergmann & Owen (2003 ▸). It was used as the background pattern for all refinements, while allowing *BGMN* a free choice of polynomial and two squared-Lorentzian peaks for required additional (Miller, 1960 ▸) background intensities. The paracrystalline fraction was composed of all of the aforementioned intensities, exceeding those of the amorphous pattern.

For the bulk crystalline fraction, the average crystallite dimensions 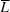 were refined anisotropically, without preferred orientation. To account for the intensity loss from Bragg reflections towards larger *s*, an isotropic thermal displacement factor *B* = 8π^2^〈*u* ^2^〉 was applied, resulting in values of the Debye–Waller factor 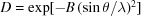 (Debye, 1913 ▸). The attenuated intensities *I*_th_ from the bulk and paracrystalline fractions *f*_bc_ and *f*_pc_ were tracked during refinement:
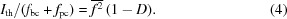



Their attribution was important to completing the paracrystalline phase scattering profiles. A portion of the intensity attenuated from the Bragg reflections is due to thermal disorder and another due to structural disorder. We determined these portions by using their attribution to minimize the gradients of the cumulative integrated ratios *f*_*i*_(*s*_p_). This is similar to Ruland’s (1961 ▸) original approach, in which the thermal factors themselves were altered to obtain constant values of the crystalline fraction when integrating over different ranges of *s*_p_.

Separating the different peak broadening contributions outlined in equation (1) is complicated by the fact that those of microstrain and paracrystallinity both increase with scattering order *n*, and therefore with scattering vector *s* = *n*/*d*. In polymers with weakly defined reflections at larger *s*, a clear differentiation between linear increases due to microstrain and quadratic increases due to paracystallinity (Hosemann & Wilke, 1968 ▸) poses a challenge. While refining both influences separately and simultaneously, we found that the starting conditions influenced the results. Reducing both into one number with a variable exponent solved this issue. Hence, we modified equation (1) to equation (5), with parameters *v* and *p*, and *n* = (*h* ^2^ + *k* ^2^ + *l* ^2^)^1/2^:
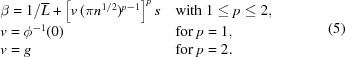
We define *v* as the disorder magnitude and *p* as the disorder indicator, *i.e.* whether it directly represents microstrain broadening ϕ^−1^(0) or paracrystalline order *g*, or portions of both. All of the above were applied according to the {*hkl*} direction. The corresponding *BGMN* structure file code section and details about the line shapes are given in the supplementary information.

From the refinement results, we calculated the individual phase fractions via equation (3), with *K* = 1, *s*_0_ = 0.57 nm^−1^ and *s*_p_ = 7.91 nm^−1^. On the basis of the work of Sao *et al.* (1997 ▸), we could then attribute a portion of the factor *B* to displacement due to paracrystalline lattice distortions via equation (6): 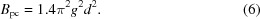
For all evaluations, care was taken to carry over the uncertainties from refinement, as well as to consider the variations between samples of the same *f*_meso_. All averaged values are medians. The corresponding uncertainties are the medians of the refinement uncertainties, to which were added the median absolute deviations of the values of individual measurements for each *f*_meso_. Errors of *f*_meso_, *i.e.* along the abscissa, result from NMR analyses of multiple synthesis batches with uniform initially weighted ratios of *R* and *S* enantiomers (Haslböck *et al.*, 2018 ▸).

## 3. Results   

### 3.1. Polymer synthesis   

All materials were obtained as white powders, except the compositions *f*_meso_ ≤ 0.6, which were clear and resin like (certainly stationary during measurements).

### 3.2. X-ray diffractometry   

Exemplary intensities from the three refined phases are shown in Fig. 1 ▸. Owing to the manner of evaluation, the progressions of the paracrystalline curves do not represent the entire scattering from the paracrystalline phase, but a convolution of scattering due to smectic arrangements and intensities attenuated from the Bragg peaks into the continuous background. The entire paracrystalline scattering includes broadening contributions to the Bragg peaks [examples given in Fig. 1 ▸of Mu (1998 ▸)]. Increasing intensities and noise at low *s* are due to the Miniflex’s variable receiving slit intensity correction. Since they occur below the range of the observed Bragg peaks, they are safe to ignore.

**Figure 2:**
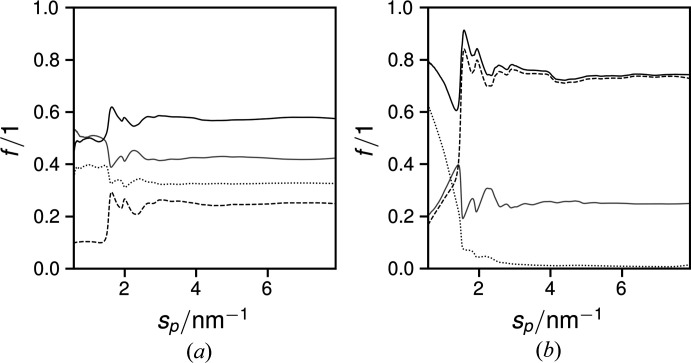
Cumulative integrated ratios for the total non-amorphous (black solid curves), amorphous (grey solid curves), bulk crystalline (dashed curves) and paracrystalline (dotted curves) intensities of samples with (*a*) *f*_meso_ = 0.64 and (*b*) *f*_meso_ = 1.00. For *f*_meso_ = 0.54, the amorphous ratio is 1.

Cumulative integration of the respective *I*_*i*_ via equation (3) led to curves of calculated phase fractions *f*_*i*_ as functions of *s*_p_ (Fig. 2 ▸). Since scattering from paracrystals transitions into a continuous curve more rapidly than that from bulk crystals (Mu, 1998 ▸), and Bragg peaks are effectively gone above *s* = 6 nm^−1^, the presented paracrystalline progressions approach the true fraction of the paracrystalline phase. Hence, all *f*_*i*_ converge on their final values, which were attained at *s*_p_ = 4 nm^−1^, corresponding to 36° 2θ. The initial *f*_pc_ in Fig. 2 ▸(*b*) is a consequence of the aforementioned intensity correction.

**Figure 3:**
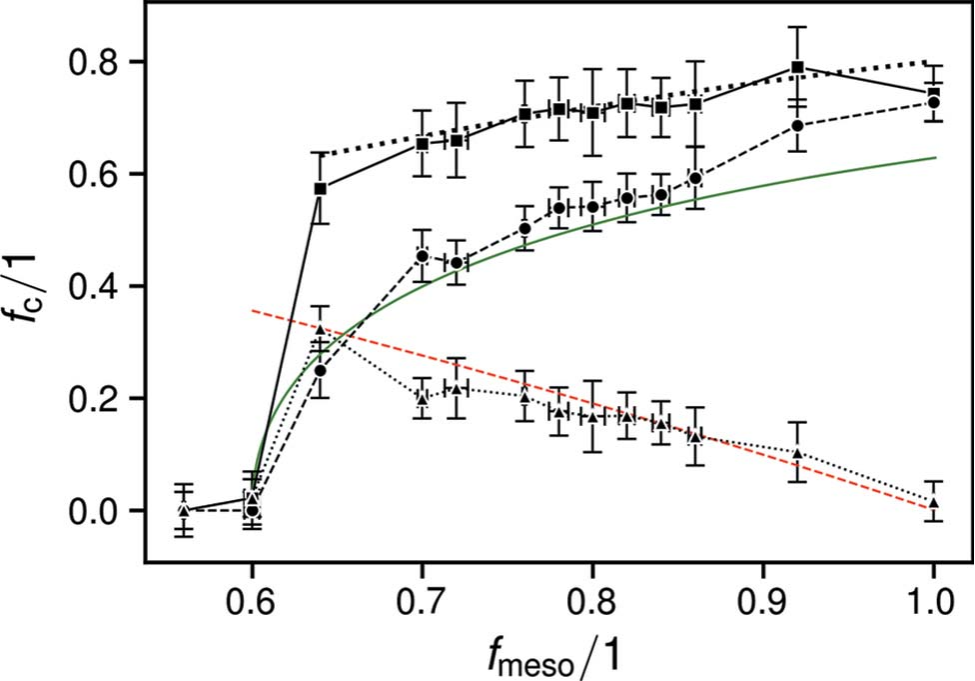
Crystalline phase fractions determined for mixed-tacticity polyhydroxy­butyrate: total non-amorphous (squares and solid lines), bulk crystalline (circles and dashed lines) and paracrystalline (upward triangles and dotted lines). The fit to previously reported values of crystallinity is given by the bold dotted curve (Haslböck *et al.*, 2018 ▸). The green solid and red dashed curves denote previously determined progressions of the exponential decay coefficients of the iso- and atactic polymer sequence length histograms, inverted, scaled and shifted simultaneously (Haslböck *et al.*, 2019 ▸).

The bulk and paracrystalline phase fractions over *f*_meso_ showed steady progressions: the former increasing logarithmically with *f*_meso_, the latter decreasing linearly (Fig. 3 ▸). For comparison, we plotted the fitting curve to the crystalline fractions determined in earlier work (Haslböck *et al.*, 2018 ▸) for the range of *f*_meso_ from which it was originally derived.

In Fig. 3 ▸, we also plotted two curves based on those from an earlier article for correlation purposes [green solid and red dashed lines in Fig. 14 of Haslböck *et al.* (2019 ▸), inverted, scaled and shifted along the abscissa]. The original curves represent the progressions of the exponential decay coefficients λ that describe the length histograms 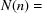 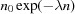 of purely isotactic and atactic polymer sequences. Here, *N*(*n*) is the number of respective sequences of length *n*, and *n*_0_ := 1 is the maximum length. The progressions of the individual λs could be recreated by equation (7), using the Lambert *W* function, the product logarithm: 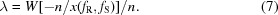
The function *x*(*f*_R_, *f*_S_) recreates an empirical relation with the fractions of *R* and *S* monomers for each *f*_meso_ (Haslböck *et al.*, 2018 ▸). For the atactic segments *x* = (*f*_R_ + 0.5)(*f*_S_ + 0.5) and for the isotactic segments *x* = *f*_R_ + 0.5*f*_R_ *f*_S_ were found to provide suitable progressions of λ with *f*_meso_ (Haslböck *et al.*, 2019 ▸). The curves shown in Fig. 3 ▸ were obtained for *n* = 0.5, inversion and by shifting the resulting curves by *f*_meso_ = 0.1 to higher values.

**Figure 4:**
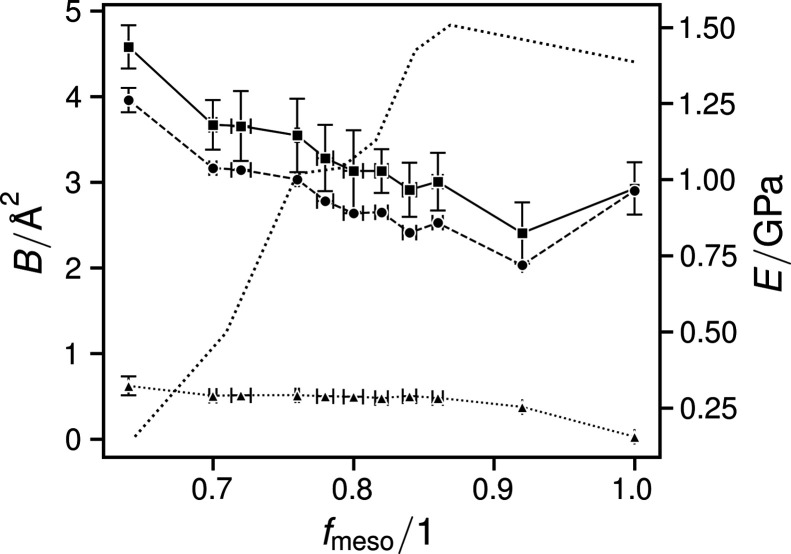
Plot of the displacement factors for the non-amorphous fractions: total (squares and solid lines), thermal (circles and dashed lines) and paracrystalline (triangles and dotted lines). The previously reported values of *E* for the entire material are given by the dotted curve (Haslböck *et al.*, 2019 ▸).

The thermal factors *B* decrease with *f*_meso_ (Fig. 4 ▸). Since the samples with *f*_meso_ ≤ 0.6 contained no bulk crystalline portion, no thermal factors were determined and they were omitted from all further presented results.

**Figure 5:**
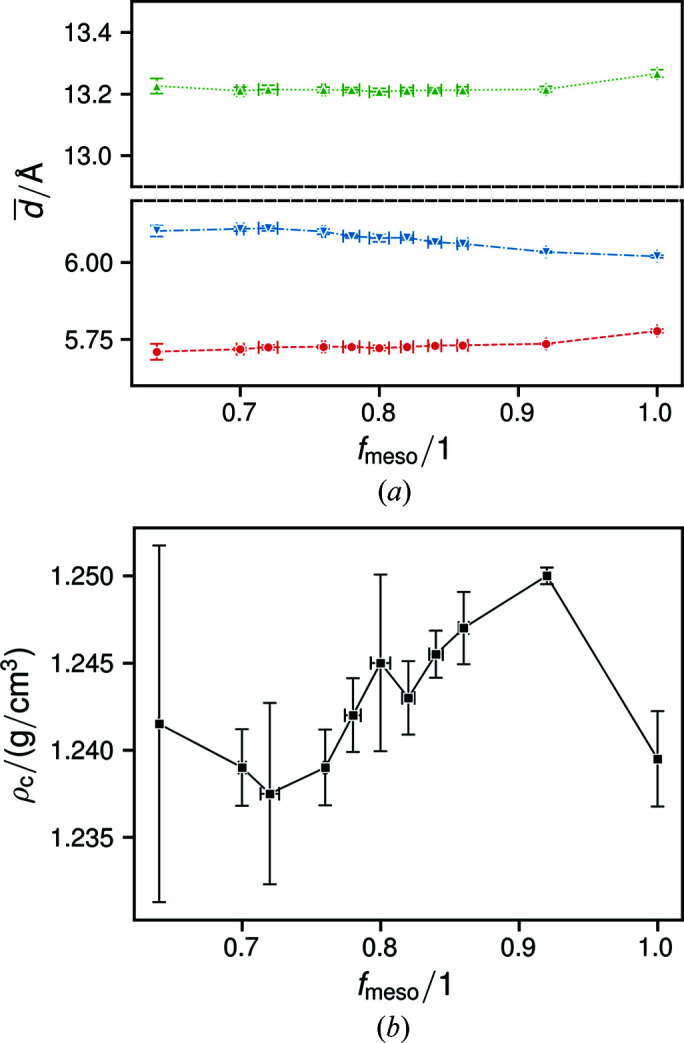
Plots of (*a*) the average lattice parameters 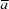 (red circles and dashed lines), 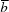 (green upward triangles and dotted lines) and 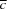 (blue downward triangles and dash–dotted lines), and (*b*) the density of the bulk crystalline phase.

As also reported earlier (Haslböck *et al.*, 2018 ▸), the lattice parameters 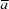 and 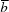 remained roughly the same, whereas 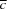 increased slightly with decreasing *f*_meso_ [Fig. 5 ▸(*a*)]. Correspondingly, the densities of the crystalline phase follow a trend towards slightly lower values with increasing tactic disturbance, noting the large error on the density at *f*_meso_ = 0.64 [Fig. 5 ▸(*b*)].

**Figure 6:**
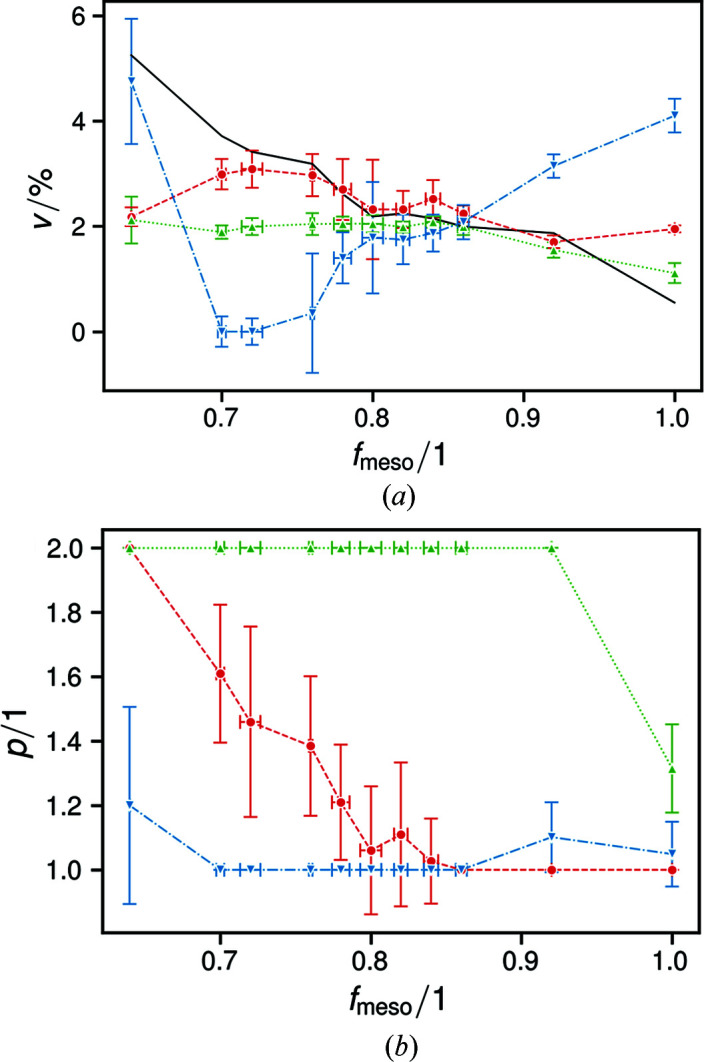
Plots of the determined disorder (*a*) magnitude and (*b*) indicator, for the directions of *a* (red circles and dashed lines), *b* (green upward triangles and dotted lines) and *c* (blue downward triangles and dash–dotted lines). The black line in (*a*) marks the progression of the total paracrystalline order magnitude *v*_pc_.

Fig. 6 ▸ shows the values of the parameters *v* and *p* for the directions *a*, *b* and *c*. The former is a measure of the magnitude of disturbance, while the latter indicates whether it signifies microstrain (Δ*d*/*d*, for *p* = 1) or paracrystalline structural [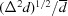, for *p* = 2] disorder.

**Figure 7:**
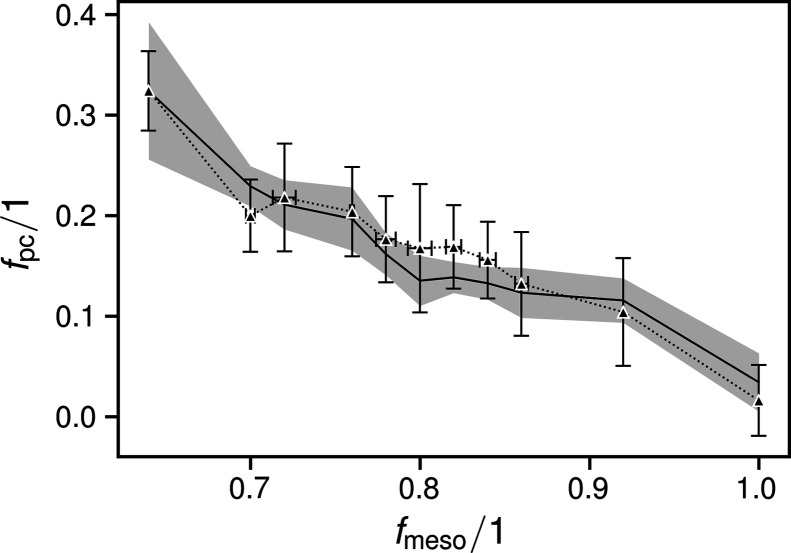
Plot of the correlations between the paracrystalline content (black upward triangles and dotted lines) and *v*_pc_ via equation (9) (full black lines with shaded uncertainties).

The paracrystalline content could be traced by calculating a single *p*-weighted paracrystalline order magnitude *v*_pc_ [equation (8) and Fig. 6 ▸(*a*)] and then matching it to the values of *f*_pc_ via equation (9) (Fig. 7 ▸): 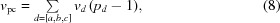


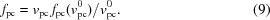




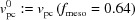
 is the data point to which we pegged the correlation. It is therefore ‘initio-quantitative’: starting from *x* (*v* ^0^), for the initial magnitude and scaling, it provides quantitative correspondence. While a theory for quantitative correlation is currently lacking, we determined that for all measurements *f*_pc_/*v*_pc_ = 6.4 ± 1.2.

## 4. Discussion   

### 4.1. Interpretation of directional disorder, considering the α-PHB structure   

In the classic crystal model, **c** is the direction longitudinal along the polymer chains (Marchessault & Kawada, 2004 ▸; Birley *et al.*, 1995 ▸). As a consequence, we determined small crystallite sizes 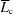 for all *f*_meso_ in earlier work (Haslböck *et al.*, 2018 ▸). In the aforementioned model, **a** and **b** are the directions perpendicular to the chain orientations. Out of the two, **a** is the direction along which single polymer chains are folded (Marchessault & Kawada, 2004 ▸; Birley *et al.*, 1995 ▸). The folded laths, already extending to their fullest in **a** and **c**, are subsequently stacked in direction **b**, forming lamellae (Marchessault & Kawada, 2004 ▸; Birley *et al.*, 1995 ▸). The stacking aspect is also supported by our own crystallite size data, which indicate that only the 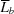 change to a significant degree with *f*_meso_ (Haslböck *et al.*, 2018 ▸). The lamella lengths in **a** are achieved by an assembly of laths ‘out of register’ towards one another. The lath edge joints interrupt the crystalline order, and hence 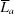 can be expected to be independent of lamella length and uniform sizes were determined previously (Haslböck *et al.*, 2018 ▸).

**Figure 8:**
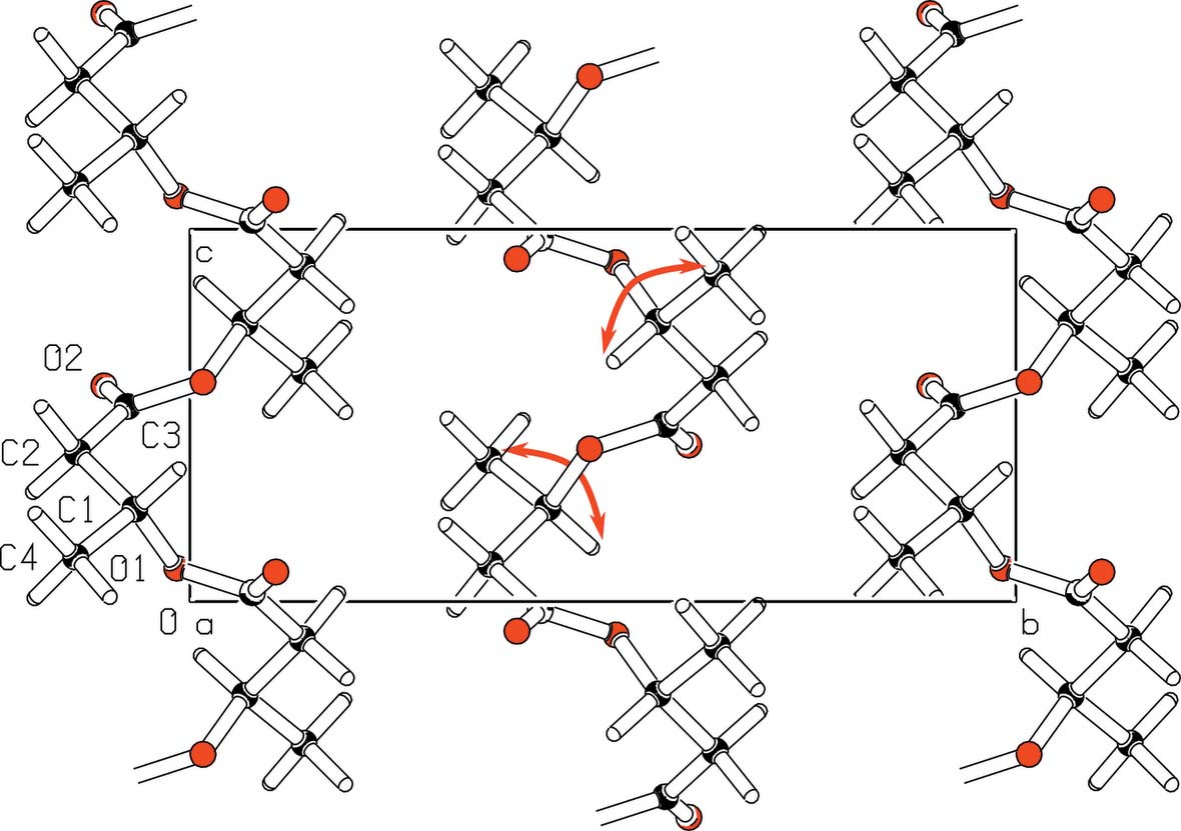
Unit cell of α-PHB viewed in lattice direction **a** with two exemplary exchanges of groups to obtain the *S* enantiomer, marked with red arrows.

In **a** and **c**, the crystallites are constituted of single polymer chains. Increasing tactic disturbance leads to unit-cell expansion in **c**, but not in **a** [Fig. 5 ▸(*a*)]. Since an observed increase of averaged lattice parameters can be caused by microstrain according to ε = 2ϕ^−1^(0) (Stokes & Wilson, 1944 ▸), it is peculiar that, while 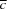 increases, *v*_*c*_ decreases (Fig. 6 ▸). If the observed expansion is not due to localized microstrain, which leads to a lattice parameter distribution ϕ^−1^(ε), then it must occur homogeneously throughout the material. Thus, the phenomenon can be traced to the structural changes when exchanging *R* monomers for their *S* enantiomers. This requires exchanging the C4 methyl group with the lone C1-bonded hydrogen (Fig. 8 ▸).

The exchange occurs roughly within a plane in the direction of **a**, along vectors ∼[011]. Since it brings the C4 methyl group into close proximity to the in-chain O1 atom, the helices along **c** are likely to be forced into a shallower angle, expanding the repeating units in this direction. In **a**, bonding between the single-chain folds is mainly due to the C4H_3_⋯O2 interaction (Wang & Tashiro, 2016 ▸). Shifting the C4 methyl group onto the other side of the chain either does not greatly alter their distance or causes the O2 atom, which projects in direction **a**, to form hydrogen bonds with other adjacent H atoms. Both would explain the weak effect of tactic disturbance on 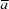.

In **b**, however, cohesion is mainly due to Van der Waals forces, requiring a close conformational match easily disturbed by tactic mismatch. It is therefore conclusive that – as indicated by *p*_*b*_ [Fig. 6 ▸(*b*)] – paracrystalline order manifests itself first in **b**, the most readily dissolved crystal direction, along which different polymer chains are joined together (Marchessault & Kawada, 2004 ▸; Birley *et al.*, 1995 ▸). Correspondingly, a typical incarnation of paracrystallinity in linear polymers is the formation of smectic structures that resemble lamellar bundles along directions perpendicular to the molecular axes (Natta & Corradini, 1960 ▸; Hosemann, 1970 ▸). This is in agreement with Hosemann’s concept of the nature of paracrystalline order, namely that former net planes are not only unevenly spaced but also distorted: bent, warped, twisted *etc*., readily envisionable among laths consisting of single polymer chains (Hosemann, 1950 ▸, 1970 ▸). Nevertheless, with increasing tactic disturbance, *p*_*a*_ increases, indicating that structural disorder of the second kind gradually manifests itself also in **a** [Fig. 6 ▸(*b*)]. At low *f*_meso_, where the ordered portions of the structure unravel completely, structural disorder manifests itself in **c** as well, leading to an increased *p*_*c*_ and, more importantly, a sudden increase in *v*_*c*_ (Fig. 6 ▸).

### 4.2. Correlation with prior electron microscopy investigations   

In earlier work, we observed branched or linear strands with diameters of 15 nm in low-voltage transmission electron micrographs of PHB with *f*_meso_ = 0.54, deposited from chloroform solution [Fig. 8 ▸ of Haslböck *et al.* (2018 ▸)]. These showed dark spots at regular intervals of 39 nm. It is our interpretation that these are of the same origin as the granular patterns observed in polyethylene crystals [Fig. 6 ▸(*b*) of Holland (1964 ▸)]. Hosemann determined that these ‘gabardine’ patterns in polyethylene, with a repeating length of 30 nm, originate from mismatch between paracrystal blocks within the image plane [Fig. 9 of Hosemann (1970 ▸)]. Since changing orientations of entire blocks would not lead to the formation of the then-observed Moiré patterns, he deduced that the orientational mismatch is due to polymer chain kinks within what appear as single crystals. These differ between blocks and therefore interrupt the lattice continuity, but conserve their lattice orientation. From crystallite size considerations, he then determined that the pattern repeating length corresponds to dimensions perpendicular to the chain orientations [Fig. 10 of Hosemann (1970 ▸)].

In our case, we note that the observed regular intervals between dark spots correspond to the crystallite dimensions 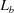 found in undisturbed PHB, at large *f*_meso_ (Haslböck *et al.*, 2018 ▸). Furthermore, we had earlier found that ‘[t]he development of the crystallite dimensions of the iso-PHB unit cell extrapolates […] to *L*_*a*_ = 15.3 nm and […] to *L*_*b*_ = 14.5 nm for *f*_meso_ = 0.5’ (Haslböck *et al.*, 2018 ▸), which corresponds to the observed strand diameters. A likely explanation is that the strands observed with the electron microscope consist of sequences of disordered paracrystal blocks with dimensions similar to those of ordered bulk crystallites.

While PHB at *f*_meso_ = 0.54 exhibited no crystalline order, either via X-ray diffraction (Haslböck *et al.*, 2018 ▸) or in thermal analyses (Haslböck *et al.*, 2019 ▸), it also did not show signs of paracrystalline ordering when measured in bulk, for this work. However, in our prior work, we presented a measurement from a thin film of *f*_meso_ = 0.54, which showed the characteristic scattering profile determined for smectic structural arrangements herein [dotted line in Fig. 1 ▸(*b*) and thin lines in Fig. 1 of Haslböck *et al.* (2018 ▸)]. Evidently, in thin films regenerated from solution, paracrystalline arrangements are formed more readily than in the bulk material. Since the transmission electron microscopy samples were also regenerated from solution, the likely explanation from the prior paragraph is indeed likely. The meaning of this finding for the bulk material is that the diminishing crystallite dimensions *L*_*b*_ with decreasing *f*_meso_ determined earlier (Haslböck *et al.*, 2018 ▸) may be interpreted as the primary building blocks retaining their dimensions, but being gradually transformed from bulk to paracrystalline order.

### 4.3. Development of fractional composition with *f*_meso_   

The values of total non-amorphous phase are within the measure of uncertainty of the values we previously reported as the fractions of the crystalline phase (Haslböck *et al.*, 2018 ▸). It is convenient that we earlier selected an integration cutoff at 35° 2θ (Haslböck *et al.*, 2018 ▸) (then based on the first halo of the amorphous pattern), since it marks the approximate point of convergence via equation (3).

Fig. 3 ▸ shows that the break in *f*_c_ and Δ*H*_m_ at *f*_meso_ ≃ 0.6, questioned in the *Introduction* [Sec sec1], exists. And, when equating *f*_c_ with the total non-amorphous phase content, it is rather sharp. However, the underlying bulk crystalline contents transition gradually to 0 along a curvature that is well described by the plotted Lambert *W* function, while the paracrystalline contents steadily increase going to *f*_meso_ = 0.6. This matches the gradual transformation of the building blocks from bulk to paracrystallinity, as proposed at the end of the previous section. Since the paracrystalline phase already incorporates directional fluid statistical order (Hosemann, 1950 ▸; Hosemann & Wilke, 1968 ▸), the transition to the amorphous phase with complete fluid-like order at *f*_meso_ = 0.6 can occur more readily than directly from the bulk crystalline phase.

As described in the *Results* [Sec sec3], the red and green curves in Fig. 3 ▸ are not equal to those presented earlier (Haslböck *et al.*, 2019 ▸). However, their qualitative progressions are good matches for the observed *f*_bc_ and *f*_pc_. At 0.64 < *f*_meso_ < 0.7, both their contents approach equal proportions, below which they are replaced by the amorphous phase. *f*_meso_ = 0.71 ± 0.05 is the value where the materials were earlier shown to exhibit maximum energies of fracture (Haslböck *et al.*, 2019 ▸). These were caused by major changes to the materials’ strengths and fracture strains, which exhibit a ‘sweet spot’ at 0.64 < *f*_meso_ < 0.7. However, the numbers of *f*_bc_ and *f*_pc_ themselves do not change suddenly; their quantities merely intersect. We therefore suspect that there is a feature of the spatial distributions of the bulk and paracrystalline phases yet unaccounted for. On the basis of the current findings, and considering the spacing of the reported data points around *f*_meso_ = 0.71 ± 0.05 (Haslböck *et al.*, 2019 ▸), we propose that in future work the mechanical properties be further refined in the range 0.64 < *f*_meso_ < 0.7.

### 4.4. Thermal factors and mechanical properties   

The paracrystalline portion of the total displacement factor expectedly increases with *f*_pc_ (Fig. 4 ▸). Their absolute values and their relation to the total *B* are supported by their similarity to the values of the ‘Ruland Parameter (*k*) and First [thermal] and Second [paracrystalline] Kinds of Distortions (*k*′ and *k*_2_) in Ramie’ cellulose, as determined by Sao *et al.* (1997 ▸). They determined near-constant values of *k*′ from untreated, heat-treated or alkali-treated mercerized cellulose (Sao *et al.*, 1997 ▸). By contrast, in this work, the thermal portion of the total displacement factor changes markedly with *f*_meso_, following the latter’s progression.

The thermal *B* is a measure of the average displacement of chain elements due to thermal motions, counteracted by their elastic bond suspension. In general, thermal factors decrease with increasing elastic parameters (Debye, 1913 ▸), allowing one to determine elastic constants *e* (Sasaki *et al.*, 2013 ▸). In earlier work, we were able to trace Young’s moduli *E*, as determined by tensile testing, with a two-phase model using the total amorphous and crystalline contents (Haslböck *et al.*, 2019 ▸). We note that the progression of *B* corresponds qualitatively and inversely to the *E* determined for the crystalline phases, which decreased to lower *f*_meso_. Remarkably, when considering the measured *E* for the entire material (Haslböck *et al.*, 2019 ▸), details that were not considered in the two-phase model can be found in the progression of *B*: the curvature at 0.75 < *f*_meso_ < 0.9 and the deviations from the respective prior trends at *f*_meso_ = 1 [Fig. 4 ▸ and supplementary Fig. S3(*b*)].

This finding, then, supports the idea that tactic disturbance ‘loosens up’ the crystal structure, leading to the reduction of the average binding strengths. This, in turn, is supported by the corresponding crystalline phase densities [Fig. 5 ▸(*b*)] and further by our qualitative observation that the solubility of mixed-tacticity PHB in chloroform increases with decreasing *f*_meso_.

### 4.5. Fitting approach   

Only by including the two additional broad peaks adjacent to the main amorphous phase peak in the refinement were we able to obtain overall plausible and consistent results. This scattering [dotted line in Fig. 1 ▸(*b*)], described in the literature as stemming from smectic arrangements, strongly resembles amorphous phase scattering, as also observed in paracrystalline (or ‘mesomorphous smectic’) arrangements of polypropylene (Natta & Corradini, 1960 ▸; Zannetti *et al.*, 1969 ▸). A portion thereof was also present in a pattern which we earlier presented as the amorphous phase pattern (Haslböck *et al.*, 2018 ▸). Owing to their similar progressions, the same results were obtained for the respective ‘crystalline’ phases earlier (Haslböck *et al.*, 2018 ▸) and ‘total non-amorphous’ phases now, as traced in Fig. 3 ▸.

As pointed out in the *Results*, a portion of the intensity in the Bragg peaks stems from the paracrystalline phase, leading to their quadratic broadening with *s* and allowing us to determine *g* via equations (1) or (5) (Mu, 1998 ▸). While the intensities attenuated from the paracrystalline phase follow a nominally different function (Vonk, 1973 ▸; Mu, 1998 ▸), the progressions of *D* for disorder of the first and second kinds are similar; examples are shown in supplementary Fig. S1. Hence, they are virtually indistinguishable from the thermal background [Fig. 4 of Hosemann (1950 ▸)].

We found that accounting for attenuation via one factor *D*, then attributing the attenuated intensities, was necessary in order to not ‘lead the algorithm’. Accounting for mixed thermal and structural attenuation directionally would – in Kavesh and Schultz’s words, and recalling that *K* wraps *D* [equation (3)] – necessitate the ‘preparation of a *K* chart for an anisotropic disorder function of two or more unequal nonzero components’, which ‘would be awkward’ (Kavesh & Schultz, 1970 ▸).

The measures of fitting quality were varied, typically with 5 < χ^2^ = (*R*_wp_/*R*_exp_)^2^ < 20 (Toby, 2006 ▸). Notably, χ^2^ increased with sample crystallinity and therefore fittable signal. This is expected by its manner of calculation and illustrates the limited absolute informative value of *R* factors (Toby, 2006 ▸). We therefore further determined that we had achieved the best sensible fit by following Toby’s conclusion that ‘the most important way to determine the quality of a Rietveld fit is by viewing the observed and calculated patterns graphically and to ensure that the model is chemically plausible’ (Toby, 2006 ▸). For comparison, we ran the refinements accounting only for micro-strain, *i.e.* omitting the program lines for paracrystalline disorder shown in the supplementary information. Then, the average χ^2^ for all refinements increased from 7.9 ± 1.2 to 8.2 ± 1.2. This illustrates the improvement of fitting quality by accounting for disorder of the second kind: minor, but evidently sufficient to yield clear results (Fig. 6 ▸).

We also compared our fitting results with prior Rietveld refinements performed on PHB. Bruckner *et al.* (1988 ▸) refined three proposed models of the α-PHB structure to recorded data, arriving at a fitting quality number for the best matching structure of 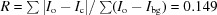. *I*_o_ is the observed, *I*_c_ the calculated and *I*_bg_ the total background intensity, for which we summed all non-bulk crystalline contributions. In the present refinements, this figure was *R*_all_ = 0.092 ± 0.004 for all assessed materials, and *R*_iso_ = 0.119 ± 0.004 for the samples composed of purely isotactic PHB.

An interesting comparison was provided by Calos & Kennard (1994 ▸), who performed refinements on freshly cast and degraded films of iso-PHB (*i.e.* with *f*_meso_ = 1) with a ‘goodness of fit’, which we assume is χ^2^, of 23.5. They, too, detected large microstrain along the *c* axis, and that ‘[t]he pattern displays [strain and thickness broadening] effects as the (020), (110) and (040) peaks stand out very sharply, whereas the general trend of all other peaks is to broaden at a greater rate as 2θ increases.’ Close inspection of their pattern ‘b’ of a degraded PHB film shows that the {020} and {040} reflections at 13.4 and 27.0° 2θ are present, the former being sharper than the latter. However, the {060} reflection at 41.0° 2θ is barely visible. It is our interpretation that this is the result of the quadratic increase of reflection broadening associated with paracrystalline order in direction **b**.

While we finally settled on the method of accounting for all intensities during a Rietveld refinement, we found Vonk’s method suitable to recreate the finally determined *f*_bc_ and *B* from the raw Bragg peak intensities (without thermal diffusive scattering) (supplementary Figs. S2 and S3) (Vonk, 1973 ▸). Since Vonk’s method includes correcting for Compton scattering, we were able to determine that the quantity of detected incoherently scattered X-rays was indeterminably small, as expected from an energy-resolving silicon strip detector.

## 5. Outlook   

We determined that, in mixed-tacticity PHB, the crystalline phase contents abruptly decrease at *f*_meso_ = 0.6. However, closer inspection revealed that this is preceded by a gradual transformation from bulk to paracrystalline order. Hence, the bulk crystalline phase contents follow a continuous progression with *f*_meso_. Around 0.64 < *f*_meso_ < 0.7, the two phases are present in approximately equal proportions. Since there the mechanical properties of PHB change in a nonlinear fashion, we consider assessing them with a smaller increment of *f*_meso_, as well as the spatial arrangements of the two crystalline phases relative to one another, an attractive undertaking.

By requiring a choice of disorder indicator from a Rietveld refinement algorithm, we were able to discriminate between directional paracrystalline order and microstrain. This allowed determining the sequence in which disorder of the second kind manifests itself directionally in mixed-tacticity PHB: **b** → **a** → **c**. A further assessment of the method’s utility, especially for non-polymeric materials, would be most interesting. In particular, quantifying the relation between the paracrystalline phase fraction *f*_pc_ and the paracrystalline order magnitude *v*_pc_ is put forward for future work.

On the other hand, the presented work required determining the intensities scattered from the paracrystalline phase by accounting for all scattered intensities. Certainly, a robust method for the calculation of intensities diverted from Bragg peaks due to the two types of disorder during refinement would be desirable for similar work.

## Supplementary Material

Supporting information file. DOI: 10.1107/S1600576720015794/fs5194sup1.pdf
