# Supplementary material for: Determining paracrystallinity in mixed-tacticity polyhydroxybutyrates
Source: J Appl Crystallogr. 2021 Feb 1;54(Pt 1):217–27. doi: 10.1107/S1600576720015794 (PMC7941308; doi:10.1107/S1600576720015794)
Supplement: Supplementary file 1 [file j-54-00217-sup1.pdf]

JOURNAL OF
APPLIED
CRYSTALLOGRAPHY

**Volume 54 (2021)**

**Supporting information for article:**

# Determining paracrystallinity in mixed-tacticity polyhydroxybutyrates

**Daniel Van Opdenbosch, Maria Haslböck and Cordt Zollfrank**

Supplementary information

*1. Verbatim BGMN structure file code to simulate microstrain and paracrystalline order.*

```
anisolin(3):sqrt(sqr(h/A)*#1+sqr(k/B)*#2+sqr(l/C)*#3)/sk //
PARAM=exph=1_1^2 PARAM=expk=1_1^2 PARAM=expl=1_1^2 //
exp=anisolin(exph,expk,expl) //
B2=k1*sqr(B1)+power(k2*power(sqr(pi)*sqrt(sqr(h)+sqr(k)+sqr(l)),exp-1),exp)*sqr(sk) //
```

Here, `h,k,l` are the Miller indices, `A,B,C` the lattice parameters, `exph,expk,expl` are the directional values of the exponent $p$, `B2` is ${\beta^2}_{hkl}$, the square of the broadening of individual reflections, `k1=1` the weighting factor for Gaussian-like broadening, `B1` the broadening due to crystallite size (proportional to $1/\overline{L}$), `sqrt(sqr(h)+sqr(k)+sqr(l))` the scattering order $n$, `sk` the scattering vector $n/d$, and `pi` $= \pi$. `k2=ANISO=ANISOSQR` is $v^2$, using a predefined tensor. In BGMN, the total sample function is then calculated by the product of one Lorentzian and one squared Lorentzian, whose half-width at half-maximum scale parameters are `B1` for the former, and `B2` for the latter; BGMN Manual Equations (5.15) to (5.18).(Bergmann & Taut, 2005)

*2. Disorder function for the second kind*

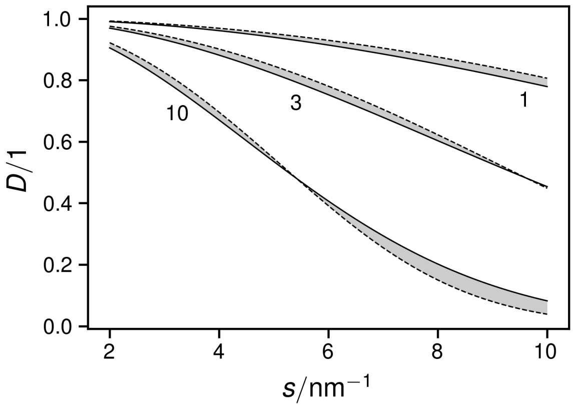


Fig. S1. Plots of disorder functions $D = \exp(-B\,(\sin\theta/\lambda)^2)$, (full lines: Thermal motions, disorder of the first kind) and $D_{\mathrm{II}} = \exp(-A\,(\sin\theta/\lambda)^2)\cdot 2/(1+\exp(-A\,(\sin\theta/\lambda)^2))$, (dashed lines: Structural disorder, of the second kind) with $A = \pi/2\cdot B$ and differences shaded grey, for $B = [10, 3, 1]$.

*3. Results from Vonk's method*

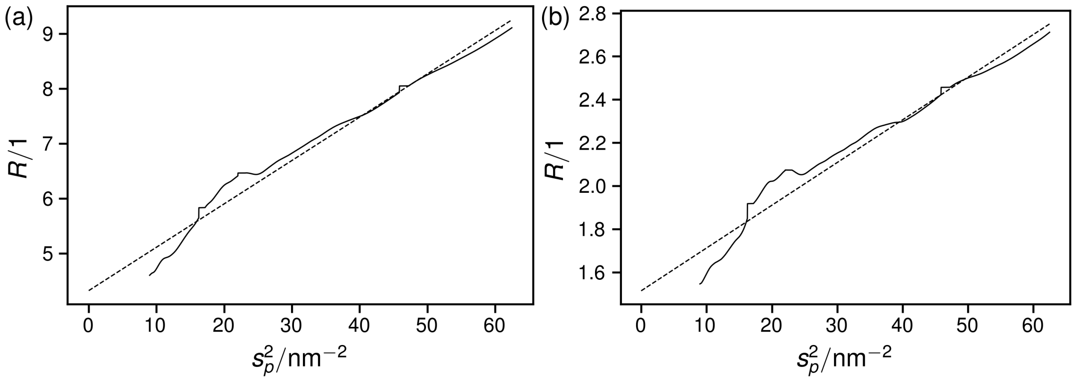


Fig. S2. Exemplary plots of $R$ (full lines) and a squared second-degree polynomial (dashed curve) over $s_p^2$ for (a) $f_{\mathrm{meso}}$ = 0.64 and (b) $f_{\mathrm{meso}}$ = 1, to visualize Vonk's method (according to Figure 1 and Equations (1-7) in cited article).(Vonk, 1973)

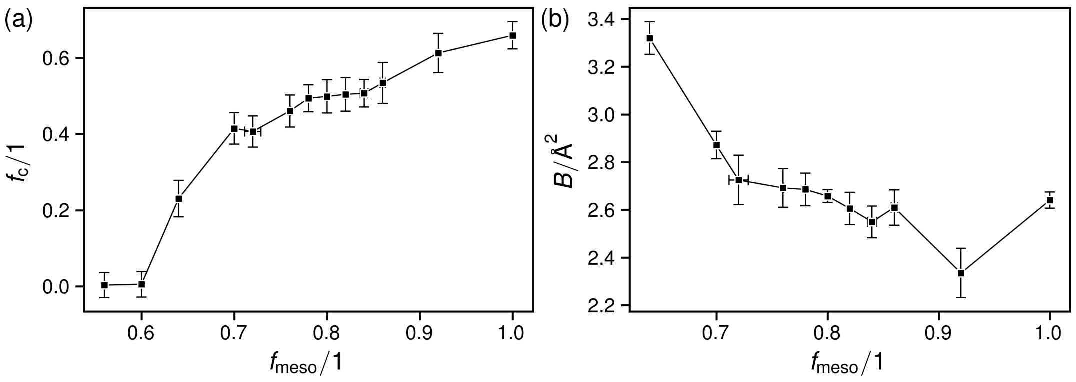


Fig. S3. (a) Bulk crystalline phase contents and (b) thermal factors determined by Vonk's method.(Vonk, 1973)
